# Sex Differences in Episodic Memory Variance

**DOI:** 10.3389/fpsyg.2020.00613

**Published:** 2020-04-17

**Authors:** Martin Asperholm, Livia van Leuven, Agneta Herlitz

**Affiliations:** Department of Clinical Neuroscience, Karolinska Institutet, Stockholm, Sweden

**Keywords:** episodic memory, variance, sex differences, mean difference, ceiling effect, meta-analysis

## Abstract

Men as a group have been shown to have larger variances than women in several areas pertaining to both biological and psychological traits, but no investigation has been performed in regard to episodic memory. We conducted an analysis on sex differences in episodic memory variance on 535 studies, representing 962,946 individuals, conducted between 1973 and 2013. Results showed that men had larger variances than women in verbal episodic memory tasks as well as episodic memory tasks having to do with spatial locations. Women, on the other hand, had larger variance than men for tasks involving remembering routes. These effects were for the most part small, and exploratory analyses suggest that they might come about, at least in part, because of measures not sufficiently controlled for ceiling effects. This means that the effects should be interpreted with caution and that further research on sex differences in episodic memory variance is needed.

## 1. Introduction

With regard to purely physical traits, it can often be shown that men and women differ largely when comparing their respective group averages. One of the most notable examples of a large difference is height where, for example, the average U.S. man is approximately 14 centimeters taller than the average U.S. woman (Fryar et al., 2016). A plethora of differences in how the average man behaves compared to the average woman have also been demonstrated when it comes to many psychological traits, such as aggression (Bettencourt and Miller, 1996), sexual behavior (Petersen and Hyde, 2010), and narcissism (Grijalva et al., 2015). Differences in mean performance between men and women have also been shown for several cognitive domains, such as mathematics, verbal skills (Maylor et al., 2007; Stoet and Geary, 2013, 2015, 2018), and spatial ability (Voyer et al., 1995).

Another such cognitive capability where sex differences in means have been shown is episodic memory, a type of memory concerning the remembrance of the what, where and when of past events that are not contained in working memory (Tulving, 2002). An example of an episodic memory would be to remember what one did yesterday, recalling the content of different events and place them in relation to one another, both in space and time. As such, episodic memory is a multifaceted concept that can be approximated in a number of ways, using different types of material to remember, for example words, images, routes, and faces. However, the most common way to assess episodic memory is by presenting a list of words, and then ask the participants to recall or recognize the earlier presented words. Asperholm et al. (2019a) showed in a large meta-analysis encompassing 617 studies, conducted between 1973 and 2013, that women, as a group, tend to outperform men on episodic memory tasks. However, the material to be remembered affected the magnitude of this advantage, with a female advantage for more verbal tasks, such as words, sentences, and prose, but also for nameable images, and locations. In contrast, there was a male advantage in more spatial tasks, such as abstract images and remembering a route. Results from this meta-analysis also indicated that the magnitude of these differences had remained stable since 1973 and that, for verbal tasks, the sex difference was somewhat smaller in childhood and old age than for other ages. Although the underlying mechanism for these sex differences are poorly understood, they have been reported in most of the examined countries (Bonsang et al., 2017; Asperholm et al., 2019b).

Sex differences are, as in the examples above, most often researched and expressed in terms of how the averages of the two sexes compare to each other. However, a somewhat overlooked aspect is to compare how the two sexes vary around the mean. This is an important aspect to consider when investigating sex differences, since it, together with the difference in means, helps to predict the ratio of men and women in the extremes. For example, a large-scale study of IQ scores among Scottish school children showed no difference between the average boy and the average girl (Deary et al., 2003). This could lead one to surmise that there also should be about as many high and low IQ boys as there are high and low IQ girls. However, the variance for boys is larger than for girls (Deary et al., 2003), thereby making boys over-represented at both extremes. On the other hand, asymmetric extremes can often also be observed in cases where there are mean sex differences present. For example, it can be seen that boys are over-represented among the top achievers in mathematical reasoning while girls have similar over-representation in verbal reasoning and writing ability, patterns that come about because of the male advantage in mathematics and the female advantage in verbal abilities (Wai et al., 2010). Thus, simply observing that there are differences in the male to female ratio in the extremes does not automatically reveal what underlying factors contribute to this. Variance is therefore an essential component to investigate in order to understand the bigger picture when it comes to sex differences.

In general, men seem to show larger variability than women. This holds true for physical traits, such as birth weight, blood parameters, and juvenile physical performance (Lehre et al., 2009). Recent pre-published data also indicate a greater male than female variance for subcortical volumes, cortical surface areas, and cortical thickness across the lifespan (Wierenga et al., 2020). The same pattern has also been shown for many cognitive abilities (Feingold, 1992b), such as verbal-, quantitative-, and figural reasoning (Lakin, 2013), as well as for regular school subjects such as mathematics, reading, and science (Hedges and Nowell, 1995; Nowell and Hedges, 1998; Machin and Pekkarinen, 2008). There are, however, also counter-examples. For example, for some progressive matrices tasks, women seem to have larger variances than men (Irwing and Lynn, 2005). Another example comes from a large population-based Romania sample, in which no consistent variance differences between men and women either on general intelligence or second-level specific, cognitive abilities were evident (Iliescu et al., 2016).

To our knowledge, only one previous study has investigated sex differences in episodic memory variance. Hedges and Nowell (1995) computed VR values (ratios of male variance to female variance) on associative memory data, a form of episodic memory, from three national U.S. surveys consisting of over 100,000 high school children. Results were inconclusive with one survey indicating larger variance for males, another larger variance for females, and a third one showing equal variances for males and females.

Why would there be sex differences in variance? One theory attempting to explain why the variance often is found to be larger for men than for women relates it to the difference in sex chromosomes (Reinhold and Engqvist, 2013). In humans, women are the homogametic sex since they have two of the same type of sex chromosome (XX) while men are the heterogametic sex because they have two types of sex chromosomes (XY). The theory states that since genetic expression is a result of the combination of genes on both chromosomes in a chromosome pair, having just a single X chromosome results in that many of the genes on it cannot be countered by corresponding genes on the less information-rich Y chromosome. This, in turn, means that mutations of a certain gene on the X chromosome for the heterogametic sex more often will be expressed, resulting in larger variance of expressions of that gene for that group compared with the homogametic sex. In humans, males happen to be the heterogametic sex, but there are also species where females are heterogametic. When comparing variance in body size, it can be shown that it is larger for the heterogametic sex irrespectively of whether that sex is male or female (Reinhold and Engqvist, 2013), in turn showing that the effect can be decoupled from the biological sex. Another theory attempting to explain why men might be more variable than women states that it is due to postnatal factors (Lehre et al., 2009). Here, it is hypothesized that men, because of their slower development, for a longer period of time are exposed to genetic expressions going in different directions. It is also theorized that their ostensibly higher sensitivity to early environmental stressors and opportunities makes them more diverse.

It is unclear to what extent any of the theories outlined above could influence sex differences in variance when it comes to human cognition, and more specifically, episodic memory. In regard to the sex chromosome theory, when examining general intelligence, genes that together are implicated for its expression are over-represented on the X chromosome compared to the Y chromosome (Zechner et al., 2001). Hence, the larger variance in males as compared to females in some cognitive abilities, including general intelligence, are at least in line with what would be hypothesized from the chromosome theory (Zechner et al., 2001; Johnson et al., 2009). However, conclusive proof in support of this theory is still missing (Giummo and Johnson, 2012; Printzlau et al., 2017).

As already noted, sex differences in means for episodic memory have been investigated in a large meta-analysis including 617 studies (Asperholm et al., 2019a), but no similar investigation has been conducted on possible differences in variances between the sexes. In order to get a fuller picture of sex differences in episodic memory, we will undertake a large-scale investigation on this topic, using the same dataset as was used in Asperholm et al. (2019a). This investigation will not only determine whether larger male than female variances exist in episodic memory, but it will also either strengthen or weaken the hypothesis that men exhibit larger variances in general as compared to women.

Since Asperholm et al. (2019a) could show that the mean sex difference in episodic memory heavily depended on the type of material to be remembered, a similar partition of the data will be analyzed here. That is, the data will be partitioned based on to what degree the task requires verbal (e.g., remembering a word list) or spatial (e.g., remembering a route) processing, in addition to categories that cannot be classified in this way, such as remembering faces or non-visual content. In addition, moderators pertaining to possible bias issues in the dataset (e.g., studies focusing on the topic of sex differences or not) will be investigated as well as age of the participants and year of publication.

In line with the evidence presented above (Feingold, 1992b; Deary et al., 2003; Lehre et al., 2009; Lakin, 2013; Reinhold and Engqvist, 2013), the overall hypothesis is that men will show more variance in episodic memory performance than women for the full dataset as well as for all material categories examined. Although the investigation performed by Hedges and Nowell (1995) on episodic memory reported conflicting results regarding sex differences in variance, there is not enough data to warrant a hypothesis going against the general pattern that has been found. Likewise, based on previous literature, there is no reason to expect that age or publication year should affect the results.

## 2. Materials and Methods

### 2.1. Dataset

The data that was used in this study has already been the basis for a meta-analysis on mean sex differences in episodic memory (Asperholm et al., 2019a). All data points in the dataset had to fulfil the following criteria with respect to the study, sample, or performance metric used: (1) the study had to be written in English, published in a peer-reviewed journal, and contain original, empirical data; (2) the sample had to consist of both males and females who had *not* been selected based on any disorder, disease, or diagnosis and who had *not* been manipulated in any way that may have affected their normal episodic memory performance; (3) the performance metric had to be based on a controlled and uniform task that was the same for all participants; (4) the performance metric had to assess episodic memory performance.

**Figure 1:**
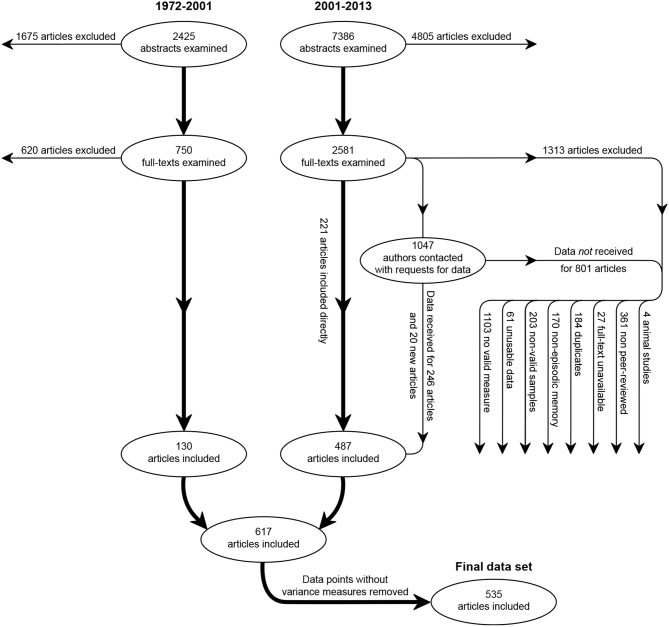
A flowchart depicting the data collection phase.

Potential articles to include were located by querying PsychINFO, PubMed, and Medline with the search terms “memory,” “humans,” and “sex or gender,” limiting the search to articles published from January 1972 to November 2013 (search queries and abstract reading sessions were conducted at two separate occasions, one starting in 2001 and one starting in 2013). This resulted in 9,811 abstracts that, after investigation, were reduced to 3,331 full-text articles that were examined. Out of these, 351 articles were included directly. If the data for a specific article was taken from a large, open database, we opted to get the data directly from the original source instead. Further, author's of 1,047 papers published after 2003 were contacted since it was judged that they might have relevant data not included in the study. This resulted in us acquiring 20 references for new articles where the data in question already were published, and receiving the necessary additional, unpublished data for 246 articles directly from the authors. All in all, this resulted in 617 studies. However, after excluding those studies which lacked variance metrics for men and women, 535 out of these 617 studies remained (see Supplementary Material for a list of these articles), representing 962,946 participants (out of which 56% were women), spanning in age range from infants to the oldest subjects being 108 years old (see Figure 1 for an overview of the data collection).

### 2.2. Moderators

A number of moderator variables pertaining to the study, sample, and task were recorded and subsequently analyzed. For task specific moderators, data points were categorized into nine categories based on the type of material to be remembered in the episodic memory task, ranging from highly verbal to highly spatial in cases where applicable. These categories were: *Verbal*—words, sentences, facts, conversations, or narrative content; *Images*—images of real or abstract objects and scenes; *Movies*—movie clips with or without sound; *Locations*—locations of objects; *Routes*—routes through space; *Faces*—images of human faces; *Sensory*—odors, tastes, and colors; *Remaining*—material that could not be placed within one of the above categories, such as composite measures based on several of them. Further, each sample was assigned an *age* value, which was defined as the middle value of the age range whenever mean age was not available. Out of the 535 studies, 467 had data making an age value possible to compute, bringing the mean of all sample ages to 48.1 years (sd = 26.9). Year comprised information regarding the year of publication.

In addition, several variables pertaining to possible bias in the dataset were defined. *Database search* indicates whether the study comes from the first (1972–2001) or second (2001–2013) database query. *Data source* indicates whether the data was retrieved directly from a published article or whether it was sent to us by authors. *Study objective* indicates whether it was explicitly stated in the article that one of the research questions was to investigate sex differences. When this was the case, the research question was almost always about difference in means, not about difference in variances. *Sampling of subjects* indicated whether the sample could be considered a population-based or convenience-based sample. A sample was considered population-based if it was indicated that the sample was randomly selected from the population.

### 2.3. Statistics

In this study, Hedges' *g* (Hedges, 1981) and the natural logarithm of the variance ratio (*lnVR*; Nakagawa et al., 2015) are used to describe mean and variance differences between two groups, respectively. Hedge's *g*, which is a common measure to describe differences in means, is computed by taking the difference in means between two groups, divide it by their pooled standard deviation, and then multiply the result by a small correction factor in order to hinder small samples from overstating the final estimate. Here, positive values mean that women outperform men and vice versa. The R package *compute.es* (Version 0.2–4; Re, 2013) was used to compute Hedge's *g* from raw values. *lnVR* is basically the natural logarithm of the ratio of two variances. Using just the ratio of two variances (*VR*) is unproblematic when computing the median variance ratio of several data points, which sometimes is done (Feingold, 1992b; Hedges and Nowell, 1995), but for most analyses and descriptive measures, a measure such as *lnVR* is needed in order to avoid giving the variance above the denominator a larger weight than the variance below the denominator in the final measure (Feingold, 1992a). However, *lnVR* can always, when analyses have been performed, be converted back to *VR* for more interpretable results, as is done in this study. Here, Equations (9) and (10) from Nakagawa et al. (2015) were used to compute *lnVR*. Positive values mean that men have larger variances than women and vice versa.

Most of the articles in the dataset contributed with several data points. This was because each article could contain several samples (e.g., age groups), where in each of these more than one episodic memory task could be tested (e.g., remembering a number of common words and remembering a number of abstract images), some of them reporting more than one dependent measure (e.g., free or cued recall; immediate or delayed recognition). To account for this hierarchical structure of the data, all analyses performed were five-level random-effects meta-analyses/meta-regressions, carried out using the *rma.mv* function in the R package *metafor* (Viechtbauer, 2010). The alpha level was always set to 0.05 for all individual analyses.

## 3. Results

First, a limited replication of the meta-analysis performed in Asperholm et al. (2019a) was carried out on the somewhat reduced dataset (535 studies instead of 617). This entailed running a meta-analysis with Hedge's *g* (with positive values indicating that women performed at a higher level than men) as the dependent variable, first without any moderators and then with material category as a moderator (see Table 1). These analyses showed similar results as in Asperholm et al. (2019a), with the categories *Movies* and *Remaining* going from being significantly different from zero to being non-significant. Results showed that women outperformed men on the categories *Verbal, Faces, Locations*, and *Sensory*, while men outperformed women on the category *Routes*.

**Table 1:** Estimated effect sizes from a meta-analysis with Hedge's *g* as the dependent variable and material category as a moderator (Omnibus *p* < 0.001; *I*^2^ = 89%).

**Type of material**	***g***	**95% CI**	***k***	***p***	***I*^2^ (%)**
Verbal	0.26	[0.24, 0.29]	333	<0.001	88
Images	0.00	[−0.04, 0.04]	174	0.98	85
Movies	0.10	[−0.03, 0.22]	19	0.13	48
Locations	0.16	[0.10, 0.21]	56	<0.001	96
Routes	-0.18	[−0.31, −0.05]	15	0.01	70
Faces	0.23	[0.15, 0.30]	47	<0.001	74
Sensory	0.32	[0.12, 0.51]	7	0.00	57
Remaining	0.07	[−0.00, 0.13]	60	0.053	68
Total	0.17	[0.15, 0.20]	535	<0.001	89

*Each row indicates whether the effect size of that specific level of the material category moderator is reliably different from 0. Estimate for Total is based on a meta-analysis using no moderators. g = Hedge's g; 95% CI = the 95% confidence interval of the estimate; k = number of studies; p = the p-value; I^2^ = statistics denoting the percentage of variation across studies that is due to heterogeneity rather than due to chance*.

**Figure 2:**
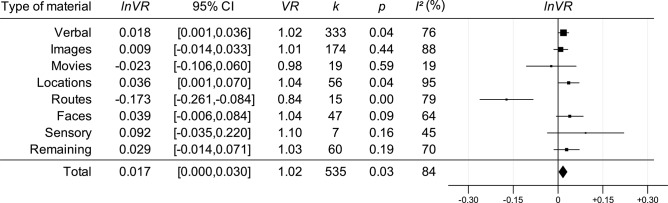
Forest plot of estimated effect sizes from a meta-analysis with *lnVR* as the dependent variable and material category as a moderator. Each row indicates whether the effect size of that specific level of the material category moderator is reliably different from 0. Estimate for *Total* is based on a meta-analysis using no moderators. *lnVR* = *lnVR*; 95% CI = the 95% confidence interval of the estimate; *k* = number of studies; *p* = the *p*-value; *I*^2^ = statistics denoting the percentage of variation across studies that is due to heterogeneity rather than due to chance.

After this, the same analyses as outlined above but with *lnVR* (with positive values indicating that men had larger variance than women) as the dependent variable were performed (see Figure 2). For the overall analysis using no moderators, the result was significantly different from zero, with men having larger variance than women. For the moderator analysis, the estimated effect sizes for the categories *Verbal, Locations* (where males had larger variance than females), and *Routes* (where females had larger variance than males) were significantly different from zero.

Next, separate meta-regressions on all material category subsets, including the *Total* dataset, were performed using age (see Table 2) and year of publication (see Table 3) as moderators. For year, no linear relationships could be found for any of the different subsets, and for age only the analyses for the *Verbal* and *Total* subsets showed significant, small, negative linear relationships, indicating that the men's larger variance became smaller across age.

**Table 2:** Best-fit intercepts and regression lines estimated from separate meta-regressions with *lnVR* as the dependent variable and age as a moderator.

**Moderator-level**		***lnVR***	**95% CI**	***k***	***p***	***I*^2^ (%)**
Verbal				296		76
	Intercept	0.094	[0.067, 0.122]		<0.001	
	Age	−0.001	[−0.002, −0.001]		<0.001	
Images				153		89
	Intercept	0.043	[−0.023, 0.116]		0.25	
	Age	−0.001	[−0.003, 0.001]		0.19	
Movies				15		38
	Intercept	0.028	[−0.133, 0.189]		0.73	
	Age	−0.003	[−0.008, 0.003]		0.35	
Locations				48		96
	Intercept	0.035	[−0.053, 0.123]		0.44	
	Age	−0.001	[−0.003, 0.002]		0.53	
Routes				14		78
	Intercept	−0.374	[−0.752,0.004]		0.053	
	Age	0.008	[−0.004, 0.020]		0.19	
Faces				41		66
	Intercept	0.025	[−0.089, 0.139]		0.67	
	Age	0.000	[−0.003, 0.004]		0.82	
Sensory				7		58
	Intercept	−0.105	[−0.403, 0.194]		0.49	
	Age	0.006	[0.000, 0.011]		0.052	
Remaining				46		62
	Intercept	−0.006	[−0.102, 0.090]		0.90	
	Age	0.000	[−0.003, 0.003]		0.87	
Total				467		85
	Intercept	0.055	[0.029, 0.081]		<0.001	
	Age	−0.001	[−0.001, −0.001]		<0.001	

*lnVR = lnVR; 95% CI = the 95% confidence interval of the estimate; k = number of studies; p = the p-value; I^2^ = statistics denoting the percentage of variation across studies that is due to heterogeneity rather than due to chance*.

**Table 3:** Best-fit intercepts and regression lines estimated from separate meta-regressions with *lnVR* as the dependent variable and publication year of the studies as a moderator.

**Moderator-level**		***lnVR***	**95% CI**	***k***	***p***	***I*^2^ (%)**
Verbal				333		75
	Intercept	−1.248	[−5.625, 3.129]		0.58	
	Year	0.001	[−0.002, 0.003]		0.57	
Images				174		88
	Intercept	3.140	[−4.821, 11.102]		0.44	
	Year	−0.002	[−0.006, 0.002]		0.44	
Movies				19		22
	Intercept	14.884	[−19.069, 48.836]		0.39	
	Year	−0.007	[−0.024, 0.010]		0.39	
Locations				56		95
	Intercept	−7.568	[−19.692, 4.557]		0.22	
	Year	0.004	[−0.002, 0.010]		0.22	
Routes				15		78
	Intercept	−31.090	[−81.667, 19.486]		0.23	
	Year	0.015	[−0.010, 0.041]		0.23	
Faces				47		63
	Intercept	11.348	[−1.046, 23.741]		0.07	
	Year	−0.006	[−0.012, 0.001]		0.07	
Sensory				7	–	–
	Intercept	–	–		–	
	Year	–	–		–	
Remaining				60		70
	Intercept	−0.823	[−13.990, 12.344]		0.90	
	Year	0.000	[−0.006, 0.007]		0.90	
Total				535		83
	Intercept	−0.960	[−4.685, 2.765]		0.61	
	Year	0.001	[−0.001, 0.002]		0.61	

*lnVR = lnVR; 95% CI = the 95% confidence interval of the estimate; k = number of studies; p = the p-value; I^2^ = statistics denoting the percentage of variation across studies that is due to heterogeneity rather than due to chance. Note that the analysis for the Sensory subset did not converge due to too little variance in the year of publication variable*.

In order to determine potential biases in the dataset, four moderator analyses were conducted with *lnVR* as the dependent variable (see Table 4): First, we compared effect sizes retrieved from the first database search (1972–2001) with those from the second (2001–2013). Second, we compared effect sizes that were retrieved directly from publications with those that were sent to us from the authors. Third, we compared effect sizes from studies where the objective in the study was to investigate sex differences and effect sizes from studies where no such objective was present. Fourth, we compared effect sizes based on whether the sampling was population-based or not. This was also the only of the analyses that had a significant omnibus test, showing that the variance sex difference was more positive (i.e., toward men having larger variance) in convenience-based samples compared with population-based samples.

**Table 4:** Estimated effect sizes from meta-analyses with *lnVR* as the dependent variable and four different moderators pertaining to possible bias.

**Moderator-level**		***lnVR***	**95% CI**	***k***	***p***	***I*^2^ (%)**
Database search				535	0.76	83
	1972–2001	0.012	[−0.023, 0.046]	91	0.51	
	2001–2013	0.018	[0.001, 0.034]	444	0.04	
Data source				535	0.71	84
	Retrieved from publications	0.019	[−0.001, 0.034]	288	0.06	
	Received from authors	0.013	[−0.009, 0.036]	247	0.24	
Study objective				535	0.14	84
	Sex differences	0.008	[−0.011, 0.026]	204	0.42	
	Other objectives	0.031	[0.007, 0.055]	331	0.01	
Sampling of subjects				522	0.03	84
	Convenience-based	0.020	[0.004, 0.036]	472	0.01	
	Population-based	−0.021	[−0.056, 0.015]	53	0.25	

*Each row indicates whether the effect size of that specific level of the moderator is reliably different from 0. Results from omnibus tests are presented on the same row as the moderator name. Estimate for Total is based on a meta-analysis using no moderators. lnVR = lnVR; 95% CI = the 95% confidence interval of the estimate; k = number of studies; p = the p-value; I^2^ = statistics denoting the percentage of variation across studies that is due to heterogeneity rather than due to chance*.

**Figure 3:**
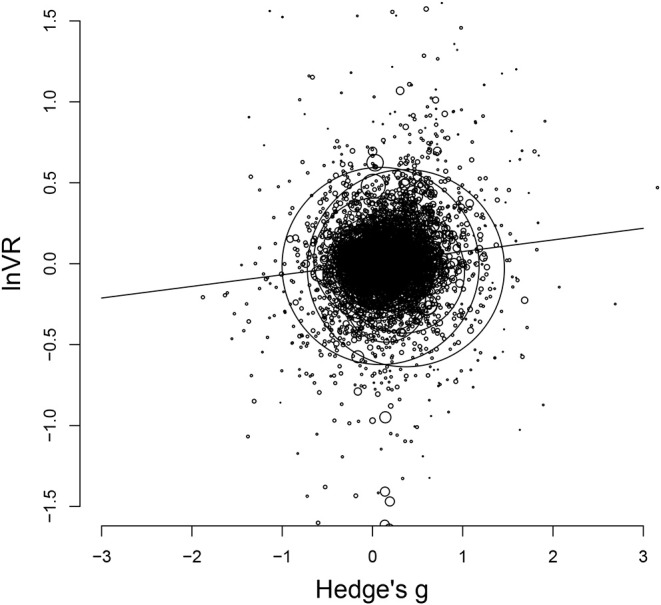
Scatterplot of *lnVR* and Hedge's *g* for the full dataset. The diameter of each data point is equal to the inverse of its squared variance. The line shows the best-fitting regression (see Table 5).

Finally, individual meta-regressions with *lnVR* as the dependent variable and Hedge's *g* as the moderator were performed as exploratory analyses for all categories (see Table 5). Results showed significant, positive relationships for *Total, Verbal, Images, Locations, Routes*, and *Faces*, and a significant, negative relationship for *Sensory*. A positive relationship here indicates that as women perform higher and higher compared to men, their variance also becomes smaller and smaller compared to the men's (and vice versa). An illustration of this effect can be seen in Figure 3 where the association is shown for the *Total* dataset.

**Table 5:** Best-fit intercepts and regression lines estimated from separate meta-regressions with *lnVR* as the dependent variable and Hedge's *g* as a moderator.

**Moderator-level**		***lnVR***	**95% CI**	***k***	***p***	***I*^2^ (%)**
Verbal				333		76
	Intercept	0.023	[0.006, 0.040]		0.01	
	Hedge's g	0.032	[0.009, 0.055]		0.01	
Images				174		88
	Intercept	0.006	[−0.029, 0.041]		0.74	
	Hedge's g	0.143	[0.097, 0.190]		< .001	
Movies				19		19
	Intercept	−0.005	[−0.058, 0.048]		0.86	
	Hedge's g	−0.030	[−0.145, 0.085]		0.61	
Locations				56		95
	Intercept	−0.012	[−0.049, 0.024]		0.51	
	Hedge's g	0.211	[0.121, 0.301]		<0.001	
Routes				15		79
	Intercept	−0.052	[−0.208, 0.104]		0.51	
	Hedge's g	0.408	[0.199, 0.618]		0.00	
Faces				47		64
	Intercept	0.006	[−0.045, 0.058]		0.81	
	Hedge's g	0.178	[0.101, 0.255]		<0.001	
Sensory				7		45
	Intercept	0.167	[−0.015, 0.349]		0.07	
	Hedge's g	−0.128	[−0.216, −0.041]		0.00	
Remaining				60		70
	Intercept	−0.006	[−0.058, 0.046]		0.83	
	Hedge's g	0.023	[−0.084, 0.130]		0.68	
Total				535		84
	Intercept	0.003	[−0.012, 0.018]		0.67	
	Hedge's g	0.072	[0.054, 0.090]		<0.001	

*lnVR = lnVR; 95% CI = the 95% confidence interval of the estimate; k = number of studies; p = the p-value; I^2^ = statistics denoting the percentage of variation across studies that is due to heterogeneity rather than due to chance*.

**Figure 4:**
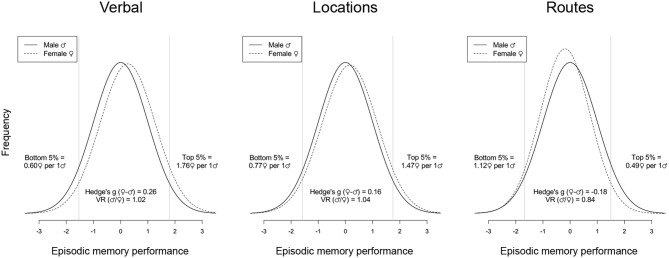
Assumed distributions of male and female performance for *Verbal, Locations*, and *Routes*. Distributions are based on the estimates of Hedge's *g* (see Table 1) and *lnVR* (see Figure 2). Only individual material categories where both estimates were significant different from zero in their respective main analyses have been plotted.

**Figure 5:**
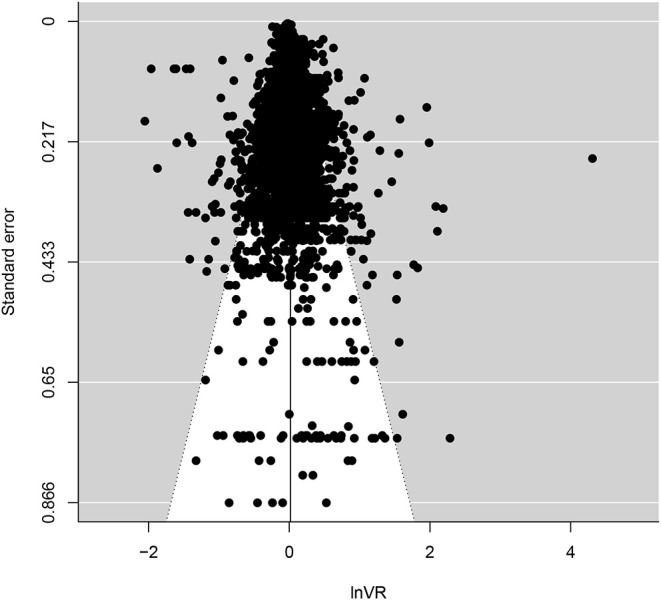
Funnel plot for the full dataset with *lnVR* on the x-axis and standard error on the y-axis. A meta-regression with *lnVR* as the dependent variable and sample size as a moderator showed no relationship between the two (Intercept: 0.017 [0.002, 0.032], *p*=.02; Sample size: 0.000 [0.000, 0.000], *p*=.30). A meta-regression with *lnVR* as the dependent variable and logged (with the natural logarithm) sample size as a moderator showed no relationship between the two either (Intercept: 0.026 [−0.017, 0.060], *p*=.28; Logged sample size: −0.001 [−0.008, 0.006], *p*=.78).

In Figure 4, assumed distributions of male and female performance have been plotted based on the estimates of Hedge's *g* (see Table 1) and *lnVR* (see Figure 2). Also, in Figure 5, a funnel plot for the full dataset is shown, and the result from a meta-regression with *lnVR* as the dependent variable and sample size/logged sample size as a moderator is reported, showing no detectable relationship between the two, indicating that the funnel plot is symmetrical.

## 4. Discussion

The goal of this study was to investigate possible sex differences in variance for episodic memory performance, where the hypothesis was that men would show larger variances than women regardless of the material to be remembered and that no variance differences would be seen for age or publication year. This was done by fitting a set of meta-analytical models on a dataset of 535 studies, previously used for a meta-analysis on sex differences in episodic memory mean performance (Asperholm et al., 2019a).

Results showed that there was a sex difference in variance for the full dataset with men having larger variances than women. Further, when running a moderator analysis with type of material to be remembered, men had significantly larger variances in both *Verbal* and *Locations*, whereas women had significantly larger variance for *Routes* (see Figure 2). No sex difference could be detected in the five remaining categories. These results are somewhat reminiscent of the inconsistency of results that Hedges and Nowell (1995) presented when investigating associative memory, where there also were examples of men being more variable, women being more variable, and no difference at all. However, our results were not in line with the hypothesis that men would be more variable regardless of material to be remembered.

Further, results showed that there were significant linear relationships between age and sex differences in variance for *Verbal* and *Total*, indicating that the men's larger variances became smaller across age. Although the reason for this finding is unclear, it should be noted that age for each sample in this analysis was rather unspecific. That is, age was computed by either taking the mean age of the sample or, alternatively, the middle value of the age range. The latter measure can differ quite substantially from the mean age and in both cases, variance around the age value was never taken into account. Further research is needed before concluding that the sex difference in variance become smaller across age in episodic memory.

It is not likely that publication bias can explain the significant sex differences in variance as the same results were found regardless of whether data were taken directly from publications or were previously unreported data obtained from authors. Similarly, whether or not there was an explicit objective of the study to investigate potential sex differences did not impact on the results. Out of the moderator analyses performed that pertained to possible bias in the dataset (see Table 4), including meta-regressions with publication year as moderator (see Table 3), it was only whether the sample was population-based or convenience-based that could be shown to have an effect. However, it is not clear why men had larger variance in convenience-based samples than in population-based samples. Again, a replication of this finding would strengthen the results.

For all of the categories where variance differences could be detected, meta-regressions showed that when the mean sex differences became larger, the variances of the excelling sex became smaller (see Table 5 and Figure 3). This could come about because of a ceiling effect, where as one sex approaches the ceiling in a certain task, their variance would also be constricted, which in turn would make their variance smaller compared to the other sex. In cases where a ceiling effect would be the result of deficiencies in how the task was constructed (for example, a word list consisting of fewer words than what some of the participants are capable of remembering), rather than because of an actual performance limit, this would contribute toward overestimating the true, underlying variance difference.

When considering the estimated *lnVR* effect sizes of the material categories (see Figure 2), it can be seen that whenever men had larger variances than women, women also outperformed men, and vice versa. This is in line with the reasoning above, where a ceiling effect would constrict the variance of the higher performing sex more. However, even if this would be the case, it is not clear whether this potential ceiling effect comes about because of methodological or functional reasons, something that would drastically affect the interpretation of the findings. This reasoning should also be viewed in the context of the reported sex differences in variances for the most part being rather small. For example, for *Verbal*, which showed the smallest effect (*VR* = 1.02), assuming equal means of the two groups would result in 1.03 men for every woman in the top/bottom 5%. Taken together, the sex differences in variance that were found (see Figure 2) should be interpreted with caution. Also, if ceiling effects indeed are affecting the outcome this would suggest that the actual sex differences in means, if anything, might be underestimated.

Investigating whether a ceiling effect contributes to the effect in other fields where sex differences in variance have been found would require a thorough examination of the underlying data. However, pointing toward a number of examples where one sex have larger variances while also performing at a higher level would at least indicate that a possible ceiling effect can *not* explain the sex differences in variance in full. Examples of this, when means and variances go in the same direction, include mathematics (Hedges and Nowell, 1995; Nowell and Hedges, 1998; Machin and Pekkarinen, 2008) and quantitative reasoning (Lakin, 2013).

A limitation of the present study is that the analyses were designed to only test for whether there are differences in variances between men and women, meaning that the results presented only can provide evidence *for* sex differences. Non-significant results only indicate that there is not enough power to detect a difference. Thus, the non-significant results can neither be used to determine that there are *no* sex differences in variance, nor to determine that differences in variances are small enough to be meaningless.

## 5. Conclusion

In summary, some support was found for the hypothesis that men have more variance than women on episodic memory tasks, but only in two out of eighth material categories investigated (*Verbal* and *Locations*), and with a reversed effect found in one of the categories (*Routes*). However, exploratory analyses indicated that these, for the most part very small, effects potentially could be exaggerated because of ceiling effects resulting from limitations in testing procedures. The result should therefore be interpreted with caution. Future research should more thoroughly investigate whether there is a ceiling effect present in fields where men and women differ in variance, and further, what the nature of this ceiling effect is. The topic of this article is an important question to elucidate since differences in variances also influences the number of men and women found at different levels of performance, even if there is a lack of mean differences between the two groups.

## Data Availability Statement

All datasets generated for this study are included in the article/Supplementary Material.

## Author Contributions

AH came up with the idea for the study. MA designed the research and carried out the analysis. MA and LL wrote the manuscript. All authors read and commented on the research.

### Conflict of Interest

The authors declare that the research was conducted in the absence of any commercial or financial relationships that could be construed as a potential conflict of interest.
