# Supplementary material for: Sex Differences in Episodic Memory Variance
Source: Front Psychol. 2020 Apr 17;11:613. doi: 10.3389/fpsyg.2020.00613 (PMC7180222; doi:10.3389/fpsyg.2020.00613)
Supplement: Supplementary file 1 [file Data_Sheet_1.PDF]

# Supplementary material

The supplementary material consists of two files:

**Effect_sizes_used_in_analyses.csv** A .csv file (which stands for *comma-separated vales* and is meant to be viewed in a spradsheet program) listing combined effect sizes for each material category within each study that was included in the analysis. Explanation of headings: Citekey = A .bib library citekey for the article that the effect size comes from (see the *Studies_included_in_analyses.bib* file for more information); *lnVR* (var) = *lnVR* and variance for a specific type of material category in a study when combining all effect sizes within it as described in Figure S1 in (Asperholm, Högman, Rafi, & Herlitz, 2019); Category = Type of material levels within each study. Levels are indicated with V (Verbal), I (Images), M (Movies), L (Locations), Ro (Routes), F (Faces), S (Sensory), and Re (Remaining); Males = The total number of males; Females = The total number of females; Samples = The total number of separate samples; Effect sizes = The total number of separate effect sizes; Published = Information on whether the sex difference data has been published (Y) or not (N).

**Studies_included_in_analyses.bib** A .bib file (which is the bibliography format used when writing LaTeXdocuments) listing bibliographical information for all citekeys mentioned in the *Effect_sizes_used_in_analyses.csv* file explained above.

# References

Asperholm, M., Högman, N., Rafi, J., & Herlitz, A. (2019). What did you do yesterday? Sex differences in episodic memory. *Psychological Bulletin*, *145*(8), 785–821. doi:10.1037/bul0000197
